# Abnormally glycosylated MUC1 establishes a positive feedback circuit of inflammatory cytokines, mediated by NF-κB p65 and EzH2, in colitis-associated cancer

**DOI:** 10.18632/oncotarget.22168

**Published:** 2017-10-27

**Authors:** Sandra Cascio, Jacque L. Faylo, Joshua C. Sciurba, Jia Xue, Sarangarajan Ranganathan, Jason J. Lohmueller, Pamela L. Beatty, Olivera J. Finn

**Affiliations:** ^1^ Department of Immunology, University of Pittsburgh School of Medicine, Pittsburgh, PA 15261, USA; ^2^ Fondazione Ri.Med, Palermo, 90133, Italy; ^3^ Department of Chemistry, University of Pittsburgh, Pittsburgh, PA 15260, USA; ^4^ Division of Pediatric Pathology, Children's Hospital of Pittsburgh, Pittsburgh, PA 15224, USA

**Keywords:** colitis-associated cancer, Mucin 1, altered glycosylation, EzH2, pro-inflammatory cytokines

## Abstract

The abnormal hypoglycosylated form of the epithelial mucin MUC1 is over-expressed in chronic inflammation and on human adenocarcinomas, suggesting its potential role in inflammation-driven tumorigenesis. The presence of human MUC1 aggravates colonic inflammation and increases tumor initiation and progression in an *in vivo* AOM/DSS mouse model of colitis-associated cancer (CAC). High expression levels of pro-inflammatory cytokines, including TNF-α and IL-6, were found in MUC1+ inflamed colon tissues. Exogenous TNF-α promoted the transcriptional activity of MUC1 as well as over-expression of its hypoglycosylated form in intestinal epithelial cells (IECs). In turn, hypoglycosylated MUC1 in IECs associated with p65 and up-regulated the expression of NF-κB-target genes encoding pro-inflammatory cytokines. Intestinal chronic inflammation also increased the expression of histone methyltransferase Enhancer of Zeste protein-2 (EzH2) and its interaction with cytokine promoters. Consequently, EzH2 was a positive regulator of MUC1 and p65-mediated IL-6 and TNF-α gene expression, and this function was not dependent on its canonical histone H3K27 methyltransferase activity. Our findings provide a mechanistic basis for already known tumorigenic role of the hypoglycosylated MUC1 in CAC, involving a transcriptional positive feedback loop of pro-inflammatory cytokines.

## INTRODUCTION

Epidemiologic and experimental evidence strongly implicates chronic colonic inflammation as one of the major risk factors for colorectal cancer (CRC). Colitis-associated cancer (CAC) can develop in the setting of inflammatory bowel disease (IBD), such as Crohn's disease and ulcerative colitis, with rapid progression, poor prognosis and high mortality rate (∼50%) [1, 2]. Similar to other solid malignancies, CAC is characterized by a robust inflammatory infiltrate and increased expression of pro-inflammatory cytokines. In sporadic CRC, inflammatory cells are recruited after the tumor is formed and participate in promoting additional mutations and epigenetic changes, whereas in CAC they seem to have a pro-tumorigenic role [2]. However, the mechanism by which chronic inflammation drives tumor initiation and progression still need to be elucidated.

In a mouse model of CAC, it was demonstrated that IKK/NF-κB pathway serves as the link between inflammation and cancer by accelerating tumor promotion and development [3]. The tumor-promoting activity of the NF-κB pathway results from its ability to induce expression of pro-inflammatory cytokines such as IL-6, TNF-α, IL-1 and IL-8 by premalignant epithelial cells and also by immune/inflammatory cells [2, 4].

MUC1 mucin is a transmembrane glycoprotein expressed at low levels on the apical surface of glandular or luminal epithelial cells. The extracellular domain of MUC1 is characterized by the presence of the Variable Number of Tandem Repeat (VNTR) region rich in serines and threonines that are heavily glycosylated with long and highly branched O-linked carbohydrates. In contrast, MUC1 is overexpressed and the VNTR is severely hypoglycosylated in all stages of development of adenocarcinoma and in various chronic inflammatory diseases including IBD [5–9]. The human and mouse MUC1 share 87% homology in the cytoplasmic tail and the transmembrane domain, whereas homology is only 34% in the VNTR region. Thus, to study the pro-inflammatory and pro-tumorigenic role of the hypoglycosylated MUC1 we used mice transgenic for the human MUC1 (MUC1.Tg). In MUC1.Tg mice, the human MUC1 promoter drives the expression of MUC1 gene in the same spatial and tissue distribution as observed in human tissues [10].

In mouse models of CAC, colon cancer is initiated by a single injection of the mutagen azoxymethane (AOM) followed by repeated oral administration of dextran sodium sulfate (DSS) to induce colonic inflammation [11]. In our previous studies, AOM/DSS treatment of MUC1.Tg mice resulted in greater weight loss, increased colon shortening and increased tumor incidence compared to WT mice [6, 12]. These results suggest that MUC1 might promote tumorigenicity by aggravating inflammation, and we set out to investigate potential molecular and cellular mechanisms underlying this function.

Here, we show that the inflammation increased expression of the hypoglycosylated form of MUC1 that could then associate with NF-κB p65 to enhance expression of pro-inflammatory cytokines in intestinal epithelial cells (IECs). This activity was mediated by Enhancer of Zeste Homolog 2 (EzH2) methyltransferase enzyme whose over-expression in many aggressive tumors is associated with poor prognosis [13, 14] and presence of distant metastases [15–19]. Moreover, it was reported that EzH2 downregulation could reduce growth of invasive breast carcinoma [15], tumor angiogenesis [20] and *in vitro* cell migration/invasion of CRC cell lines [13, 21]. EzH2 is the catalytic core of Polycomb repressive complex 2 (PRC2) that silences gene transcription through trimethylation of histone 3 lysine 27 (H3K27me3), known as a repressive epigenetic marker [22]. EzH2 also recruits a DNA methyltransferase that further enhances gene repression [23]. Although EzH2 was initially discovered as a component of a transcriptional repressor complex, recent studies show that EzH2 is able to activate gene transcription by forming transcriptional complexes through mechanisms that do not involve histone methylation [24–26]. We demonstrate here that over-expression of aberrant MUC1 glycoform amplifies the inflammatory signal induced by AOM/DSS treatment and establishes a positive regulation of inflammatory cytokines, mediated by p65 and EzH2, which positively controls tumor growth and progression.

## RESULTS

### The presence of human MUC1 increases AOM/DSS-induced colonic inflammation and colitis-associated tumorigenesis

**Figure 1:**
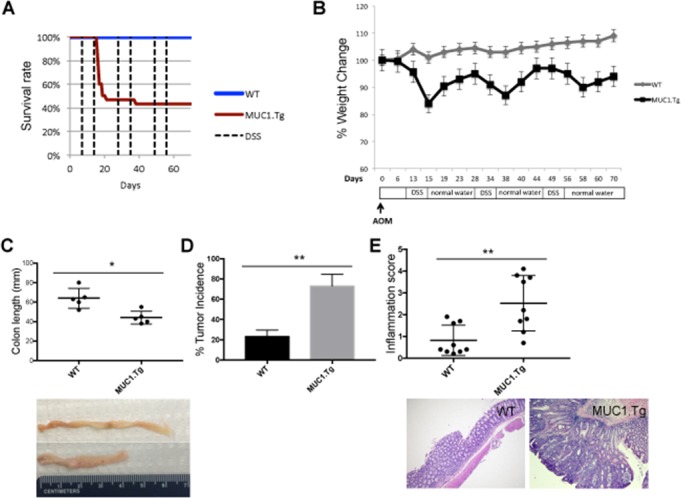
MUC1 promotes inflammation and tumorigenesis in AOM/DSS-treated mice WT and MUC1.Tg mice were given an AOM i.p. injection followed by three cycles of 1.2% DSS in drinking water, as described in Materials and Methods. **(A)** Kaplan-Meier survival curves of WT (n = 24) and MUC1.Tg (n = 31) mice during AOM/DSS treatment. **(B)** Body weight of WT and MUC1.Tg following AOM/DSS treatment. **(C)** Colon length of WT (n= 9) and MUC1.Tg (n=12) mice. Picture is an example of one WT and one MUC1.Tg mouse. Measurement of colon lengths in sacrificed mice at day 72. **(D)** Incidence of tumors in the colons. Statistical analysis was carried out with unpaired t test with Welch's correction ^**^ indicates *P*<0.05. **(E)** *Upper Panel*: Results of histological scoring of sections from WT and MUC1.Tg mice. The data shown are representative of three independent experiments; *Lower Panel*: Hematoxylin and eosin staining. Histopathology of colon tissues of DSS/AOM treated mice. An example of a WT mouse showing colon inflammation while MUC1.Tg mice developed colon adenocarcinomas.

We have previously shown that MUC1.Tg mice were exquisitely sensitive to injury when exposed to 2% DSS for 7 days [12]. Using this treatment regimen, MUC1.Tg mice developed rapid and severe clinical disease characterized by weight loss and bloody diarrhea with high mortality rate (80%) after the second cycle of DSS. Here we applied a modified protocol that includes a shorter cycle of treatment with lower DSS concentration. Figure 1 shows that a single intraperitoneal (i.p.) injection of 10 mg/kg AOM given to WT and MUC1.Tg mice (n= 24 and 31, respectively), followed by three cycles of 1.2% DSS in drinking water for five days, caused no mortality in WT mice whereas the mortality rate in MUC1.Tg mice was around 55% (Figure 1A). Weight loss in WT mice reached a maximum of 5% followed by a complete recovery. In MUC1.Tg mice, weight loss was more severe, with a maximum loss of 18% two days after DSS treatment, and incomplete recovery. At the time of sacrifice, 70 days after AOM injection, MUC1.Tg mice were still 9% below their initial weight (Figure 1B). Rectal bleeding, diarrhea and colon shortening, a macroscopic parameters of colitis severity, were more pronounced in MUC1.Tg mice compared to WT (Figure 1C). Importantly, no difference in body weight and colon length was detected between WT and MUC1.Tg mice without treatment (Data not shown). Even though distal colonic tumors developed in both WT and MUC1.Tg mice, the incidence of tumors was twice as high in MUC1.Tg mice compared to WT mice (Figure 1D). H&E-stained sections showed that MUC1.Tg mice developed adenomas with high-grade dysplasia and higher inflammation score (Figure 1E) compared to WT mice.

### Exogenous pro-inflammatory cytokines increase overall MUC1 expression and its hypoglycosylated form in epithelial colon cells

**Figure 2:**
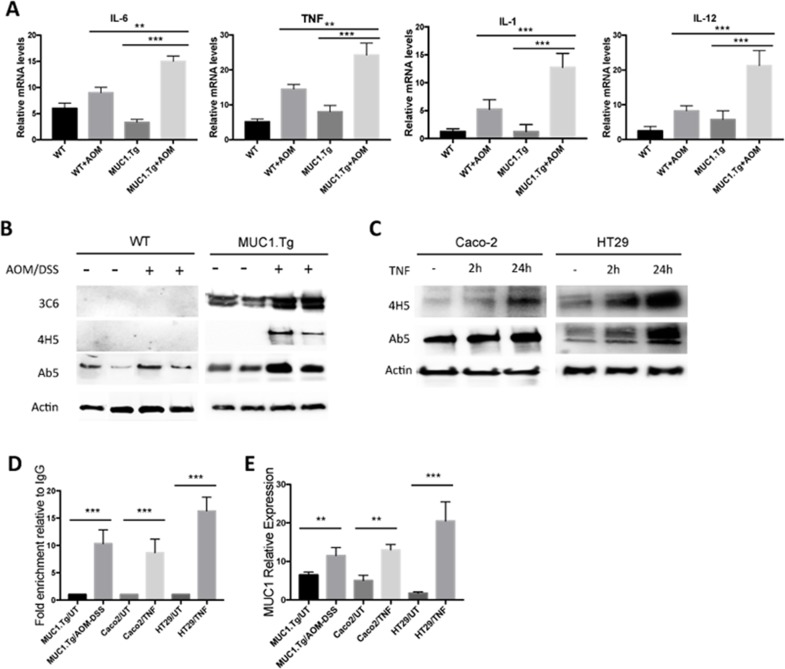
Chronic Inflammation promotes MUC1 expression via NF-κB activation **(A)** Detection of mRNA levels by real-time PCR in the indicated colonic tissues. Gene expression of each target molecule was normalized to GAPDH. Statistical analysis was carried out with One-way Anova. ^**^ or ^***^ indicate *P*<0.05 or P < 0.001 respectively. **(B)** Western blotting of whole cell lysates from IECs isolated from untreated or AOM/DSS-treated WT and MUC1.Tg mice with anti-MUC1 VU-3C6, VU-4H5 or Ab5 antibodies. Actin was used as loading control. **(C)** SDS-PAGE gel of Caco-2 and HT-29 cells treated with TNF-α or left untreated and then blotted with indicated antibodies. **(D)** ChIP assay. Soluble chromatin was immunoprecipitated with anti-NF-κB p65 antibody and analyzed by qPCR for NF-κB consensus sites on MUC1 promoter. ChIP was performed on IECs isolated from colon tissues of MUC1.Tg mice treated with AOM/DSS or on TNF-treated Caco-2 and HT-29 cells. Quantification of binding was represented as fold-enrichment relative to IgG. ^***^ indicates P < 0.001. **(E)** Detection of mRNA levels of MUC1 by real-time PCR. Gene expression was normalized to GAPDH. Statistical analysis was carried out with One-way Anova. ^**^ or ^***^ indicate *P*<0.05 or P < 0.001 respectively.

An inflamed microenvironment facilitates cell proliferation and neoplastic transformation of intestinal epithelial cells (IECs). Pro-inflammatory cytokines, such as IL-1, IL-6 and TNF-α, are associated with colitis-associated cancer [27]. First, we measured pro-inflammatory cytokine expression by real-time PCR (RT-PCR) in the inflamed colon tissues of mice. We found that IL-6, TNF-α, IL-1 and IL-12 were significantly increased in AOM/DSS-treated MUC1.Tg mice by 2.23, 1.8, 2.5 and 3.3 fold, respectively, compared to WT mice (Figure 2A). No significant difference in cytokine expression was detected between untreated MUC1.Tg and WT mice.

Next, we investigated whether high levels of inflammatory cytokines could affect the expression of MUC1 in epithelial colon cells and, consequently, enhance MUC1-dependent intracellular signaling. To detect MUC1 protein expression in AOM/DSS-treated mice we performed Western blotting using the anti-MUC1 Ab5 antibody that recognizes the cytoplasmic tail domain of both murine and human MUC1 proteins (Figure 2B). Human MUC1 was detected by using anti-VU 3C6 (Figure 2B) specific for the GVTSAPDTRPAP epitope of the VNTR region [28]. Because the expression of hypoglycosylated MUC1 has been associated with highly inflamed colon tissues and all stages of adenocarcinoma [12, 29, 30], we also analyzed the expression of human hypoglycosylated MUC1 using the VU-4H5 antibody that specifically detects non-glycosylated PDTRP epitope (Figure 2B). As expected, all inflamed colon tissues of AOM/DSS-treated MUC1.Tg mice showed higher levels of total and hypoglycosylated MUC1 compared to untreated MUC1.Tg mice. To investigate whether there was a direct effect of pro-inflammatory cytokines, in particular TNF-α, on MUC1 expression and its abnormal glycosylation, we performed a series of *in vitro* experiments. Caco-2 and HT-29 human colon cancer cells were stimulated with TNF-α at 50 ng/ml for 2h and 24h. The expression of total MUC1 and its hypoglycosylated form increased in a time-dependent manner (Figure 2C).

It was previously reported that the region of the MUC1 promoter between −600 and −400 is important for maximal MUC1 transcription [31, 32]. Located within this transcriptional region, at -503/−495 and -589/−580, are specific consensus sites for NF-κB p65 (GGGRNNYYCC, where R is purine, Y is pyrimidine, and N is any base). While MUC1 transcriptional regulation by cytokines that signal via NF-κB factors in breast cancer cells is well documented [33], the role of NF-κB in regulating MUC1 gene expression in colon cancer cells has not been determined. We assessed that function using the Chromatin Immunoprecipitation (ChIP) Assay. As shown in Figure 2D, after AOM/DSS treatment, the MUC1 promoter region containing both κB sites, displayed p65 occupancy. We also confirmed the interaction of p65 with MUC1 promoter in Caco-2 and HT-29 cells treated with TNF-α (Figure 2D). Additionally, compared to untreated MUC1.Tg mice, the MUC1 mRNA was significantly up-regulated after AOM/DSS treatment (Figure 2E). Similar data were obtained in human colon cancer cells Caco-2 and HT-29 treated with TNF- α (Figure 2E). In AOM/DSS-treated mice, no significant changes were detected in Muc1 expression (Figure 2B). In addition, ChIP assay revealed a very weak association of p65 on Muc1 mouse promoter (Data not shown).

### AOM/DSS treatment increases expression of NF-κB-dependent pro-inflammatory cytokines in intestinal epithelial cells of MUC1.Tg mice

**Figure 3:**
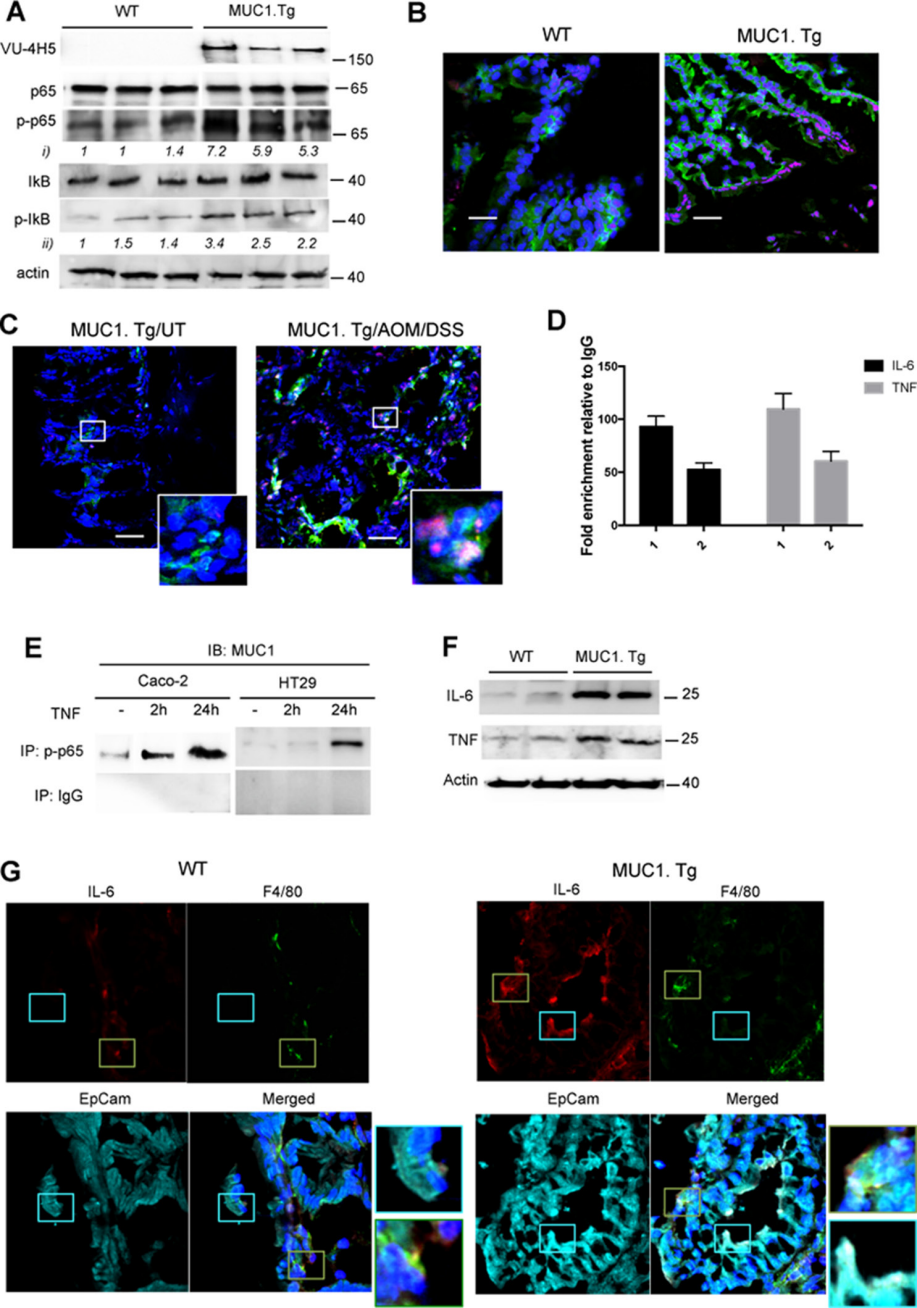
MUC1 promotes expression of NF-κB-dependent pro-inflammatory cytokines in IECs after AOM/DSS administration **(A)** Western blotting of whole cell lysates from IECs isolated from WT and MUC1.Tg mice with anti-MUC1 VU-4H5, anti-phospho-p65, total p65, total IκB and anti-phospho-IκB antibodies. Actin was used as loading control. Densitometry analyses were performed with ImageJ software and anti-pp65 (i) or anti-pIκB (ii). Band intensities were normalized to β-actin and quantified with respect to one control mouse set to 1.0. Note: the blotting representing anti-MUC1-VU4H5 Ab staining was partially used in Figure 2B. **(B)** Confocal double-stainingimmunofluorescence microscopy analysis of phospho-p65 (red) and phospho-IκB (green) expression in colons of AOM/DSS-treated WT and MUC1.Tg mice. Nuclei were stained with DAPI (blue); Bar: 100 μm. **(C)** Confocal double-stainingimmunofluorescence microscopy analysis of phospho-p65 (red) and hypoglycosylated MUC1 (green) expression in colons of AOM/DSS-treated MUC1.Tg mice or left untreated. (UT). Nuclei were stained with DAPI (blue); Bar: 100 μm. **(D)** ChIP assay. Soluble chromatin from MUC1+IECs was immunoprecipitated with anti-p65 (1) and anti-MUC1 Ab5 (2) antibodies and then analyzed for the proximal region of IL-6 and TNF-α promoters. Quantification of binding was represented as fold-enrichment relative to IgG. **(E)** Co-Immunoprecipitation Assay. Immunoprecipitatetd NF-κB p65 nuclear proteins from indicated cells were immunoblotted with anti-MUC1 Ab5 antibody. **(F)** IECs lysates immunoblotted with anti-IL-6 and anti-TNF-α antibodies. **(G)** Confocal immunofluorescence microscopy of frozen colon tissue samples, fixed and stained with F4/80 (green), EpCAM (cyan) and anti-IL-6 antibody (red). Magnification 80X.

We have previously demonstrated that hypoglycosylated MUC1 in human breast cancer directly associates with NF-κB family members [34] and acts to amplify NF-κB-regulated cytokine expression [35–38]. Therefore we assessed whether the hypoglycosylated MUC1 might do the same in our mouse model of CAC. Higher expression of phospho (276)-p65 and phospho-IκBα was seen in colons of AOM/DSS treated MUC1.Tg mice compared to control WT mice both by Western blotting (Figure 3A) and by confocal immunofluorescence (Figure 3B). Total p65 and IkB were detected as controls (Figure 3A). Confocal analysis showed that nuclear co-localization of MUC1 with NF-κB p65 occurred in IECs of MUC1.Tg mice (Figure 3C). Next, we performed ChIP assay to assess the enrichment of p65 (1) and MUC1 (2) on IL-6 and TNF-α promoters (Figure 3D). The interaction between p65 and MUC1 was also confirmed by co-immunoprecipitation assay in TNF-stimulated Caco-2 and HT-29 cells, precipitating with anti-phospho-p65 antibody and immunoblotting with anti-MUC1 Ab5 antibody (Figure 3E). Similar to our previously reported data in breast cancer cells [34], we found that nuclear MUC1 directly associated with NF-κB p65 and occupied the promoter of NF-κB regulated genes.

In order to assess the source of IL-6 and TNF-α in AOM/DSS treated WT and MUC1.Tg mice we performed Western blotting analyses (Figure 3F) and confocal immunofluorescence microscopy (Figure 3G). In the latter, F4/80 and EpCAM were used as specific markers of macrophages and epithelial cells respectively. Our results revealed that IL-6 (Figure 3F and 3G) and TNF-α (Figure 3F and Supplementary Figure 1) in WT mice were produced by macrophages while in treated MUC1.Tg mice these inflammatory cytokines were produced by both macrophages and the colonic epithelial cells.

### Inflammatory microenvironment enhances EzH2 expression in AOM/DSS-treated colon tissues of MUC1.Tg mice

**Figure 4:**
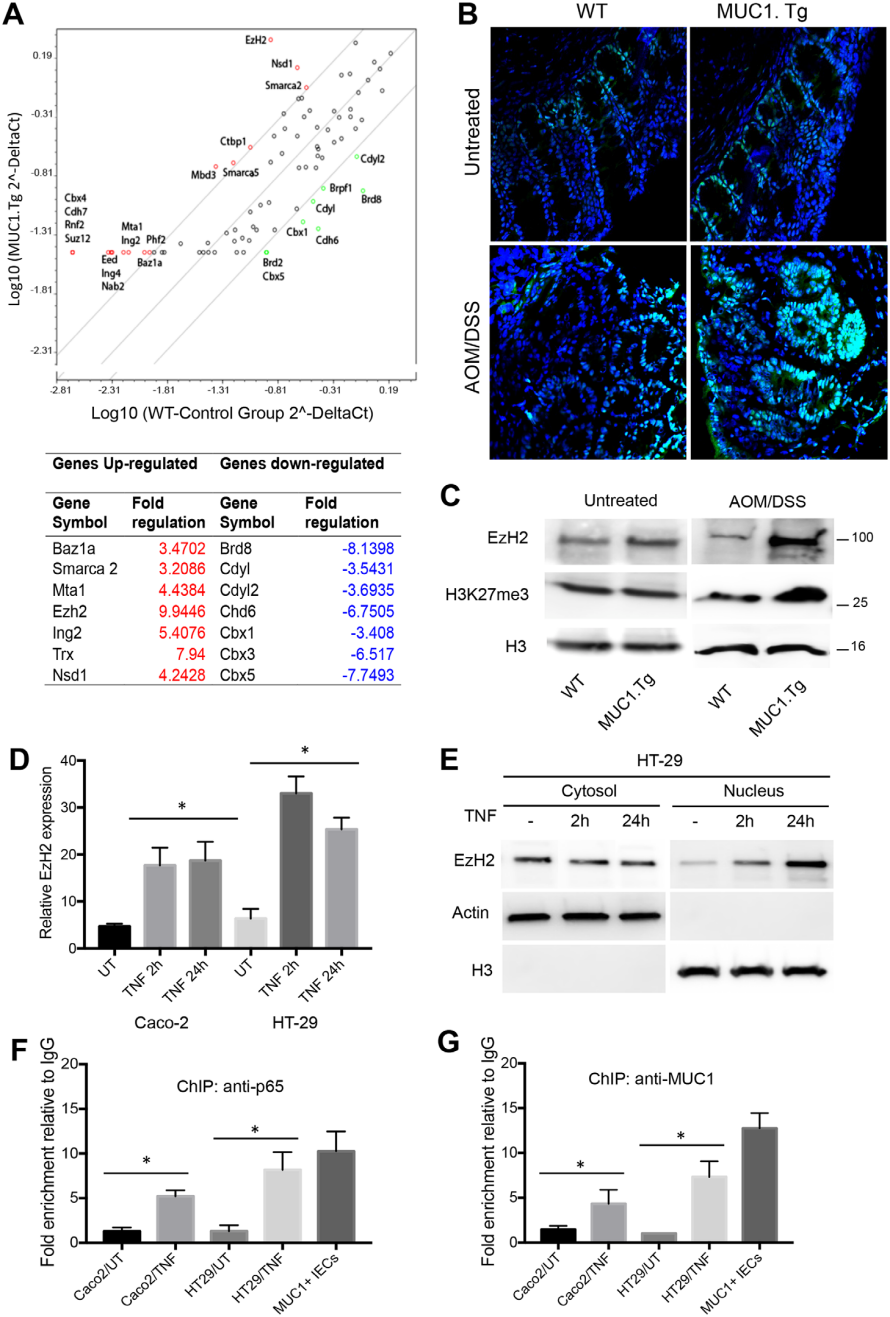
AOM/DSS-treatment up-regulated EzH2 expression via NF-κB pathway activation in IECs of MUC1.Tg **(A)** Eighty-four cancer related genes were analyzed using RT^2^ Profiler™ PCR Array. Scatter plot of the hybridization intensity of each gene in the two groups: WT (x-axis-group 1) and MUC1.Tg (y-axis- group 2). The middle line indicates a fold-change (2-ΔCt) of 1. The top and the bottom lines indicate the fold-change in gene expression threshold. The two points under the bottom line represent downregulated genes. Significant genes with fold change <±3.2 and p-values <0.05. **(B)** Confocal immunofluorescence microscopy of frozen colon tissue fixed and stained with anti-EzH2 antibody (green); Nuclei were stained with DAPI (blue). **(C)** Nuclear proteins were immunoblotted with anti-EzH2, anti-H3K27m3 antibodies. Histone H3 was used as loading control. **(D)** EzH2 mRNA levels and **(E)** cytosolic and nuclear protein expression were analyzed by Real-Time PCR or by Western blotting in indicated cells stimulated with TNF for 2h or 24 hours or left untreated. **(F-G)** ChIP assay was performed in TNF-stimulated or unstimulated Caco-2 or HT-29 cells and in IECs isolated from AOM/DSS-treated MUC1.Tg mice. Soluble chromatin was immunoprecipitated with NF-κB p65 (F) and MUC1 Ab5 (G) antibodies and then analyzed by qPCR for kB1 consensus site on promoters of EzH2. Quantification of binding was represented as fold-enrichment relative to IgG. Statistical analysis was carried out with unpaired One-way ANOVA. ^*^ indicates *P*<0.05.

To gain insight into the mechanisms by which MUC1 contributed to the regulation of IL-6 and TNF-α expression, we investigated epigenetic modifications occurring on their promoters. Total RNA from colons of AOM/DSS-treated MUC1.Tg and WT mice was analyzed with RT^2^ Profiler PCR Array Mouse Epigenetic Chromatin Remodeling Factors as described in Material and Methods. Among the 84 genes in the PCR array, 18 genes were up-regulated and 8 down-regulated by at least 3.2 fold (Figure 4A). One of the genes up-regulated in MUC1.Tg mice was EzH2 that belongs to the Polycomb-repressive complex 2 and commonly functions by inducing (tri) methylation of H3K27me3. Other up-regulated genes were Nds1 and Trx1 responsible for the methylation of H3K36me and H3K4me, respectively. In contrast, chromobox domain (Cbx) 1, 3 and 5 genes were found to be down-regulated in treated MUC1.Tg mice. They encode adaptor proteins that recognize a specific motif sequence (ARKS) of repressive marks, including histone H3 trimethylated at Lys9 (H3K9me3) and Lys27 (H3K27me3) [39, 40].

Confirming this result, we found by immunofluorescence staining (Figure 4B) and Western blotting (Figure 4C) over-expression of EzH2 in colon tissues of AOM/DSS-treated MUC1.Tg compared to WT mice. No difference in EzH2 expression was detected in untreated MUC1.Tg and WT mice (Figure 4B and 4C). To determine whether high levels of EzH2 were induced by inflammatory stimuli, we analyzed EzH2 mRNA and protein levels in TNF-stimulated Caco-2 and HT-29 cells. EzH2 mRNA (Figure 4D) and protein (Figure 4E) expression started to increase after 2hrs of TNF-α stimulation and remained high at 24 hours. Notably, EzH2 abundance remained markedly high in the nucleus after a prolonged stimulation with TNF-α. Analysis of EzH2 proximal promoter region revealed that three NF-κB sites are located at -574/566, -179/−160 and −102/−93, which we termed κB1, κB2 and κB3, respectively. ChIP assay indicated that after TNF-α stimulation there was an increased in association of p65 with the κB1 site of EzH2 promoter (Figure 4F). The enrichment of p65 on EzH2 promoter was also confirmed in AOM/DSS-treated MUC1.Tg IECs (Figure 4F). Treatment with TNF-α did not result in significant changes in p65 association with κB2 and κB3 sites of the EzH2 promoter (Data not shown). Since MUC1 interacts with p65 (Figure 3C and 3E), we performed a ChIP assay to assess the interaction of MUC1 with EzH2 promoter. A strong association was detected between anti-MUC1 Ab5 antibody with κB1 on EzH2 promoter (Figure 4G).

### EzH2 positively regulates transcription of IL-6 and TNF-α independently of its methyltransferase activity

**Figure 5:**
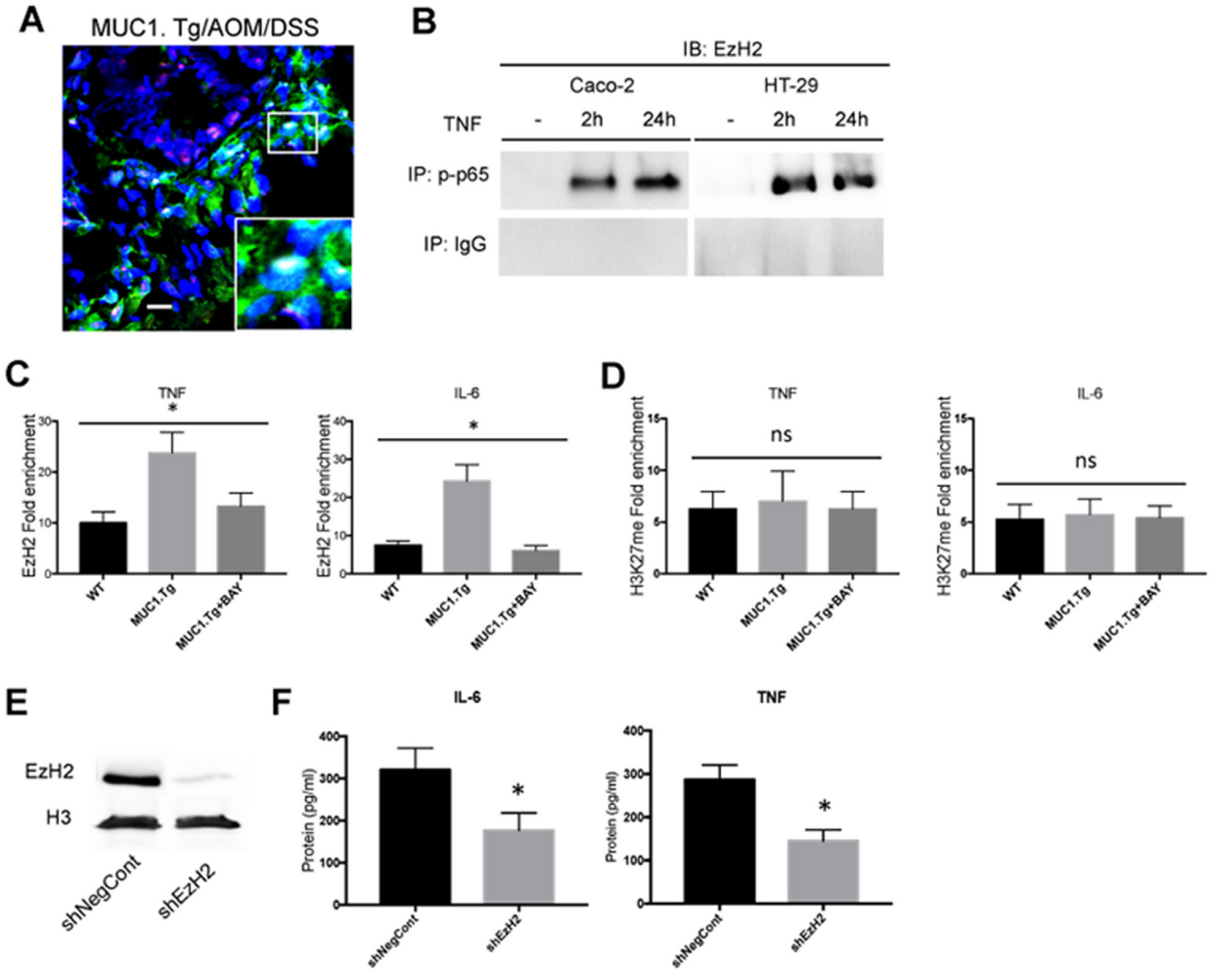
EzH2 regulates inflammatory cytokines expression in MUC1+ IECs from AOM/DSS-treated mice **(A)** Confocal immunofluorescence microscopy of frozen colon tissue samples, fixed and stained with anti-NF-κB p65 (red) or EzH2 (green) antibodies. Bar: 100 μm. **(B)** Co-Immunoprecipitation Assay. NF-kB, p65 immunoprecipitated nuclear proteins from indicated cells were immunoblotted with anti-EzH2 antibody. **(C-D)** ChIP assay: soluble chromatin was immunoprecipitated with indicated antibodies and analyzed for κB consensus sites of IL-6 and TNF-α promoters. Quantification of binding was represented as fold-enrichment relative to IgG. **(E)** Western blotting of whole cell lysates of IECs transfected with an shRNA targeting EzH2 (shEzH2) or a target control (shNegCont), as described in Material and Methods, with anti-EzH2 antibody. Actin was used as loading control. **(F)** Indicated cytokine production by IECs transfected with shEzH2 RNA or target control (shNegCont). N=6 per group. Statistical analysis was carried out with one-way ANOVA, ^*^ indicates *P* < 0.05, ns= not significantly different.

It was demonstrated recently that in breast cancer, EzH2 promotes a constitutive activation of NF-κB that leads to an increase in its target genes expression, including IL-8, IL-6 and TNF-α cytokines [24], suggesting that it might be doing the same in our model of CAC. A nuclear association between EzH2 and p-p65 was detected in colon tissues of AOM/DSS-treated MUC1.Tg mice by confocal microscopy (Figure 5A) and by co-immunoprecipitation in TNF-stimulated Caco-2 and HT-29 cells (Figure 5B). We analyzed the binding of EzH2 to IL-6 and TNF-α promoters by ChIP assay in IECs using primer pairs spanning the promoter regions containing the consensus NF-κB-binding sites of IL-6 (−75 to −63, GGGATTTTCC) [41], and TNF-α (−510 to −488, GGGGCTTTCC) [35]. Although EzH2 was found on IL-6 and TNF-α promoters of IECs isolated from both MUC1.Tg and WT mice, this association was stronger in MUC1.Tg mice (Figure 5C). To determine whether the association of EzH2 with the chromatin on IL-6 and TNF-α promoters in MUC1.Tg mice was modulated by NF-κB p65, IECs were cultured and treated with BAY-117085, a specific inhibitor of IκB phosphorylation (Supplementary Figure 2A). The amount of EzH2 on TNF-α and IL-6 promoters was significantly reduced (Figure 5C). The same experiments were performed in Caco-2 cells. Similarly, inhibition of NF-κB pathway by BAY-117085 drastically reduced EzH2 on the IL-6 and TNF-α promoters (Supplementary Figure 2B).

EzH2 regulates gene expression by catalyzing H3K27 trimethylation [42]. We asked whether such catalytic function is required for EzH2 to positively regulate MUC1 and p65-dependent cytokine transcription in our mouse model of CAC. As shown in Figure 4C, H3K27me3 expression levels were higher in AOM/DSS-treated IECs of MUC1.Tg when compared to IECs of AOM/DSS-treated WT mice. However, despite the EzH2 enrichment, no corresponding H3K27m3 enrichment was detected on IL-6 and TNF-α promoters of IECs of MUC1.Tg mice compared to WT mice (Figure 5D). Moreover, the treatment of IECs isolated from inflamed tissues of MUC1.Tg mice with BAY-117085, did not affect the association of H3K27me3 with IL-6 and TNF-α promoters (Figure 5D). There was also no detectable difference between BAY-117085-treated Caco-2 cells and untreated cells (Supplementary Figure 2C). To further confirm that EzH2 regulates TNF-α and IL-6 expression independently of its methyltrafserase activity, we treated IECs cells with GSK-126, an inhibitor of the catalytic site of EzH2 (Supplementary Figure 2D). GSK-126 treatment did not affect the expression levels of both TNF-α and IL-6 in IECs isolated from AOM/DSS-treated MUC1.Tg (Supplementary Figure 2E).

It has been shown that EzH2 down-regulation can reduce growth of invasive breast carcinoma [15–17], tumor angiogenesis [20], cell migration/invasion and metastasis in colorectal cancer cells [13, 21]. However, nothing is known about the effect of EzH2 blockade on inflammatory cytokine expression in inflamed intestinal cells that could promote tumorigenesis. To explore this potential function, IECs of AOM/DSS treated MUC1.Tg mice were isolated and transfected with shRNA targeting EzH2, as described in Materials and Methods. EzH2 shRNAs caused nearly complete reduction of EzH2 protein expression compared to cells transfected with control shRNA (Figure 5E). After treatment of IECs with EzH2 shRNA, the expression of IL-6 and TNF-α was measured by ELISA (Figure 5F) and Real-Time PCR (Data not shown). Down-regulation of EzH2 by RNA interference significantly reduced the expression of TNF-α and IL-6.

### Co-expression of MUC1, phospho-p65 and EzH2 in human colon cancer tissues

**Figure 6:**
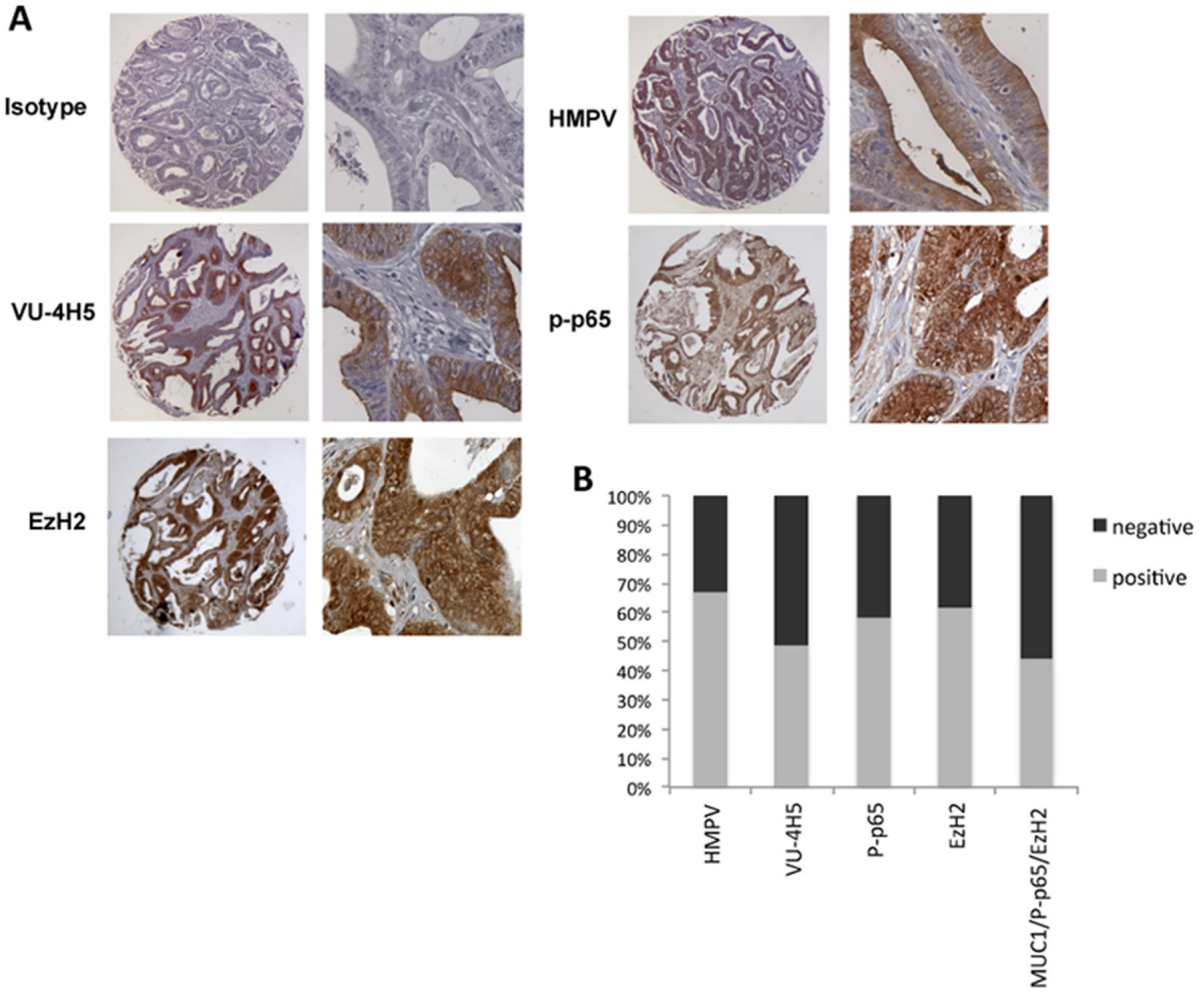
Co-expression of hypoglycosylated MUC1 with p-p65 and EzH2 in colon adenocarcinoma **(A)** Immunohistochemistry staining of the indicated proteins in colon adenocarcinoma. Original magnification is 200X. **(B)** Quantification of the percent of colon adenocarcinoma expressing total MUC1 (HMPV), hypoglycosylated MUC1 (VU-4H5), p-p65 (276) and EzH2 in colon adenocarcinoma (n=70).

We have reported that the hypoglycosylated form of MUC1 is over-expressed in colon cancer and its expression is associated with advanced grade of tumors [12]. Immunohistochemical analyses have also shown that EzH2 is significantly overexpressed in colon cancers when compared to adjacent normal tissue or benign colon adenoma [14, 21, 43]. Moreover, activation of NF-κB pathway and phosphorylation of p65 is a hallmark of inflammation with constitutive NF-κB pathway activity found in many different cancers including colon cancer [3, 38]. We were interested in the degree of co-expression of EzH2, p-p65 and MUC1 in colon cancer compared to a healthy colon or benign disease. We performed immunohistochemistry on tissues microarrays (TMA) containing 4 normal, 16 benign (including polyps and adenoma) and 55 malignant cases, with each sample in duplicate. Serial tissue sections were stained separately with primary antibodies specific for the hypoglycosylated VNTR of MUC1, phopsho (276)-p65 and EzH2. Normal tissues showed no hypoglycosylated MUC1 expression and no or only weak expression of phopsho-p65 and EzH2. Tissue samples of polyps or adenomas showed variable intensity of hypoglycosylated MUC1, phospho-p65 and EzH2 expression, from very low to moderately high. However, all samples from low to high-grade adenocarcinoma revealed strong expression of tumor form of MUC1, phopsho-p65 and EzH2 (Figure 6A and 6B). Notably, 90% of hypoglycosylated MUC1 positive tissues were also positive for expression of both phospho-p65 and EzH2 (P<0.001). Figure 6A shows a representative staining of an adenocarcinoma sample overexpressing hypoglycosylated MUC1, phospho-p65 and EzH2. Staining with anti-MUC1 HMPV antibody that recognizes the VNTR region independently of the glycosylation status of MUC1 was used as control.

## DISCUSSION

In this study, we demonstrated that hypoglycosylated MUC1, by induction and direct association with NF-κB p65, contributed to the up-regulated TNF-α and IL-6 expression in inflamed and tumorigenic IECs. We also observed an essential role of EzH2 in regulating MUC1- and NF-κB p65-dependent gene expression in CAC.

The AOM/DSS murine model of CAC represents a valuable model to study the role of inflammation in colon carcinogenesis. The increased colon inflammation and tumor incidence detected in MUC1.Tg mice compared to WT mice suggested a key role of human MUC1 in controlling inflammation-driven cancer. Alignment of murine and human MUC1 genes revealed significant homologies centered on the transmembrane and cytoplasmic tail domains whereas homology is only 34% in the tandem repeats of the extracellular domain [44]. Therefore, MUC1 extracellular domain was responsible for the severe chronic inflammation and higher tumor incidence detected in our MUC1.Tg mice subject to CAC.

The increased expression of MUC1 in inflamed colon tissues suggested an inflammation-dependent up-regulation of MUC1 in IECs. Our mechanistic studies revealed that recombinant TNF-α induced MUC1 gene expression in human colon cancer cells through activation of NF-κB pathway and association of p65 to MUC1 κB promoter sites. We also observed over-expression of the abnormally glycosylated form of MUC1. Altered glycan profile has been found in patients with active colitis and associated with the degree of inflammation and also with the severity of disease course [45, 46]. Inflammatory cytokines are involved in O-glycosylation alterations, especially sialylation and sulfation, in several chronic inflammatory pathologies, including cystic fibrosis, inflammatory bowel diseases and CAC. Likely, cytokines-driven inflammation modulates mucin glycosylation changes by regulating the expression of glycosyltransferases involved in the biosynthesis of glycan chains [47, 48]. However, we are currently investigating the specific molecular events involved in inflammation-induced MUC1 glycosylation alterations in CAC.

In response to the AOM/DSS treatment, colon tissues of MUC1.Tg mice showed high levels of IL-1 and TNF-α, two cytokines that trigger the canonical NF-κB pathway. In agreement with previous studies [34, 49], we found that human MUC1 directly associated with NF-κB p65 and lead to an increase in expression of NF-κB regulated genes.

Here, we also showed that NF-κB pathway is implicated in the activation and recruitment of EzH2 to the chromatin. We observed nuclear association between MUC1, p65 and EzH2 and their co-occupancy on IL-6 and TNF-α promoters of MUC1^+^ IECs. Our findings further indicated that EzH2 promotes the transcription of TNF-α and IL-6 independently of its methyltransferase activity on lys27 of Histone 3. EzH2 was discovered as repressor transcription factor, component of the PRC2 complex, known to induce trimethylation of H3K27me. However, other non-enzymatic transcriptional activator functions of EzH2 have been observed in some triple negative breast and prostate cancers [50].

Therefore, the hypoglycosylated form of MUC1, through p65 activation, is involved in epigenetic regulation of inflammatory cytokines enhancing their expression. This result is in line with recent studies showing that MUC1, also via NF-κB signaling, induces the expression of methyltrasnsferase DNMT1 and thereby upregulates DNA methylation in acute myeloid leukemia (AML) [51].

In mouse models of IBD and CAC, we have previously detected extensive accumulation of pro-inflammatory cell populations in MUC1.Tg mice at the sites of inflammation as well as in the tumor microenvironment [8, 12]. Analysis of human colon cancer tissues microarray showed that hypoglycosylated MUC1, phospho-p65 and EzH2 are co-expressed in colon adenocarcinoma. Given that TNF-α and IL-6 are implicated in human IBD and CAC [2, 52, 53], MUC1/p65/EzH2 may represent a new signaling axis that controls their expression not only in mice but also in human CAC.

Tumor forms of MUC1 are chemotactic to immature dendritic and inhibit their ability to stimulate type 1 helper T cell responses [54]. Moreover, the tumor form MUC1-ST, though the engagement of Siglec-9, induces macrophage differentiation to a TAM-like phenotype [55]. Thus, we propose that the MUC1-induced pro-inflammatory cytokines expression in IECs attracts inflammatory cells, especially mononuclear cells, from the venous system to the tumor site. Once mononuclear cells differentiate into macrophages and/or dendritic cells, they are a further source of inflammatory cytokines that create a pro-tumor microenvironment and accelerate tumor growth and progression. Thus, the newly identified MUC1-p65-EzH2 axis might create the favorable microenvironment for tumor initiation and progression.

## MATERIALS AND METHODS

### Animals and tumor induction

C57BL/6 mice were purchased from the Jackson Laboratory (Bar Harbor, ME, USA).MUC1 transgenic mice were originally purchased from Dr. S.J. Gendler (The Mayo Clinic, Scottsdale, AZ, USA). A transgenic construct containing the entire human MUC1 gene sequence, and 1.5 kb of 5′ sequence and 800 bases of 3′ sequence, was injected into fertilized C57BL/6 mouse eggs [10]. C57BL/6 and MUC1.Tg are bred at the University of Pittsburgh (Pittsburgh, PA, USA). To induce colitis-associated cancer, 8-10 week-old mice Wild Type (WT) and MUC1.Tg were injected i.p. with 10 mg/kg AOM and kept on regular water for 7 days. After 7 days, mice received three 5-day cycles of 1.2% DSS in drinking water, followed by 14 days of sterilized tap water. At the completion of each DSS cycle, mice were monitored for weight loss. 70 days after AOM administration, mice were sacrificed, colons were measured and tumors were counted. Sections were analyzed for the degree of inflammation. Experiments were conducted with cohorts of 6-8 mice WT and MUC1.Tg mice. All experiments were repeated three times and approved by the Institutional Animal Care and Use Committee of the University of Pittsburgh.

### Isolation and culture of intestinal epithelial cells (IECs) and cancer cell lines

Colons were opened longitudinally and washed with Ca+ and Mg+ free Hank's balanced salt solution (HBSS) to remove feces and debris. Colons were then cut in small pieces and incubated in HBSS containing 0.145 mg/ml DTT, 5 mM EDTA, 1 M HEPES, 10% FBS and 1% penicillin/strep at 37 C for 20 min for 2 cycles. IECs were filtered through a 40-μm cell strainer (BD) and centrifuged at 1200 rpm for 5 min at 4C. After three washes with PBS, IECs were cultured at 37 C in complete DMEM (Dulbecco's Modified Eagle Medium, Cellgro, Mediatech, Inc., Herndon, VA, USA) medium in 6-well plates. HT-29 and Caco-2 human colon cancer cell lines were grown in RPMI-1640 (Royal Park Memorial Institute Medium, Cellgro, Mediatech, Inc.) medium supplemented with 10% heat-inactivated fetal bovine serum, 100 units/mL penicillin, 100 μg/mL streptomycin and 2 mmol/L L-glutamine. For specific experiments cells were activated with 100 ng/ml TNF-α (R&D Systems, Minneapolis, MN). 10 μM BAY 11-7085 (Calbiochem, Billerica, MA, USA) was used to inhibit the NF-κB pathway. 200 nM GSK-126 (Millipore) was used to inhibit the EzH2 methyltransferase activity.

### Plasmids and transfection

EzH2 shRNA and Negative Control plasmids were purchased from Santa Cruz. Plasmid transfection was performed with Lipofectamine 2000 (Invitrogene Life Technology) according to manufacturer's instruction.

### Immunofluorescence confocal microscopy

Frozen mice colon tissues were fixed in 4% paraformaldehyde for 20 min and permeabilized in 0.5% Triton-X100 for 20 min. The fixed cells were incubated with anti-IL-6, anti-TNF-α (Abcam), EpCAM (Santa Cruz), anti-phospho(276)-p65, anti-phoshp-IκB, anti-EzH2 (Cell Signaling), anti-IL-6 (Abcam) for 16h at 4C followed by secondary anti-mouse Alexa-488 or Cy3 antibody (Invitrogen Life Technology) for 1h at RT. Nuclei were stained with mounting medium with DAPI (VectorLab).

### Immunohistochemistry

Human colon cancer tissue paraffin sections (catalog number Z7020032) were purchased from Biochain (Newark, CA, USA). Slides were deparaffinized by baking overnight at 59°C. Endogenous peroxidase activity was eliminated by treatment with 30% H_2_O_2_ for 15 min at room temperature. Antigen retrieval was performed by microwave heating in 0.1% citrate buffer. Nonspecific binding sites were blocked with Protein Blocking Agent (Thermo Fisher Scientific). The anti-MUC1 Ab VU4H5, which recognizes the epitope APDTRPAP in the VNTR region of the hypoglycosylated MUC1, was purchased from Santa Cruz Biotechnology. The anti-phospho(276)-p65 and anti-EzH2Ab were purchased from Cell Signaling. Staining was performed by the avidin-biotin-peroxidase complex method with a commercial kit (Vectastain ABC kit; Vector Laboratories, Burlingame, CA, USA). Positive signals were visualized by a DAB Kit (BD Pharmingen, San Jose, CA, USA). The total inflammation score for each sample was determined as previously described [8].

### Analysis of protein expression

Segments of the distal colon of both WT and MUC1.Tg mice treated AOM/DSS, were minced and suspended in 2 ml of the following lysis buffer: 150 mM NaCl, 50 mM Tris-HCl pH 7.5, 2 mM EDTA, 1% NP-40 and complete protease inhibitor cocktail (Roche). Samples were then sonicated and passed through 70 um strainer. Protein extracts were purified through centrifugation (300 g, 15′, +4°C) and supernatants were stored at −80°C. IL-6 and TNF-α proteins were measured by ELISA according to the manufacturer's protocol (Biolegend, San Diego, CA, USA).

For Western Blotting we used total proteins or fractionated proteins from IECs and human colon cancer cell lines. Total cell proteins were extracted using RIPA buffer (150 mM NaCl, 0.5 sodium deoxycholate, 0.1% SDS, 1% NP-40 and 50 mM Tris-HCl) with commercial protease inhibitors (Complete Protease Inhibitor Cocktail from Roche, Mannheim, Germany) and phosphatase inhibitors (Phosphatase Inhibitor Cocktail II, Sigma-Aldrich, St Louis, MO, USA). Cytosolic and nuclear proteins were isolated using NE-PER Nuclear and Cytoplasmic Extraction Kit from Thermo Scientific and following the manufacture's instructions. Proteins were separated by SDS-PAGE and transferred to PVDF membranes. Blots were incubated with the following antibodies: anti-EzH2, H3K27me3, anti-phospho IκB, anti-phospho-p65(276) (Cell Signaling), anti-actin, anti-MUC1 VU-4H5; anti-p65, anti-IκB, anti-H3 (Santa Cruz Biotechnology, Santa Cruz, CA, USA), anti-MUC1 3C6 (a gift from Dr. Hilgers, Free University, Brussels, Belgium).

### Chromatin immunoprecipitation assay

Chromatin Immunoprecipitation Assay was performed on the IECs utilizing the commercially available EpiQuick High Sensitivity ChIp Kit P-2027 (Epigenetek, Farmingdale, NY), according to the manufacturer's instructions. Tissues were fixed with 1% formaldehyde for 10 min at 37 C, followed by glycine stop-fix solution. We used 4 μg of the following antibodies: anti-EZH2 (Millipore), anti-H3K27me3 (Epigentek), anti-phospho p65 (276) (Santa Cruz), anti-MUC1 Ab5 (Thermo Fisher Scientific).

The presence of IL-6 and TNF-α gene promoter sequences in immunoprecipitated DNA was identified by 34 cycles of PCR using the primer sequences published previously [34]. In control samples, primary antibody was replaced with non-immune IgG. All experiments were repeated at least three times.

### Quantitative real-time PCR

Total RNA was extracted from colon tissues of treated and untreated mice using an RNeasy mini kit (Qiagen,) according to the manufacturer's instructions. A total of 2 μg of RNA was reverse-transcribed using a SuperScript first strand kit (Invitrogen). A total of 4 μl of RT products was used to amplify IL-6 and TNF-α and GAPDH as an internal control. Real-time PCR was performed using a SYBR Green PCR kit (Qiagen) and a StepOnePlus realtime PCR system (Applied Biosystems). The sequence of each primer has been described before [34].

### Gene expression profiling of epigenetic chromatin remodeling factors

Gene expression profiling of Mouse Epigenetic Chromatin Remodeling Factors was performed using the RT^2^ Profiler PCR Array PAMM-086ZA (Qiagen). PCR array is a 96-well plate containing RT2 Profiler PCR Primer Assays for a set of 84 related genes, plus five housekeeping genes and three controls. Data analyses were performed using the web-based analysis software (http://www.sabiosciences.com/pcrarraydataanalysis.php). For each PCR reaction, the Excel sheet calculated two normalized average cycle threshold (Ct) values, a paired t-test p-value and a fold-change. PCR array quantification was based on the Ct number. Fold-change and fold-regulation values >2 were indicative of upregulated gene; fold-change values <0.5 and fold-regulation values <-2 were indicative of downregulated genes. Results were expressed as the mean values ± standard deviation and the p-values were calculated based on a Student's t-test of the replicate 2-ΔCt values for each gene in the MUC1.Tg group and WT control group. A p-value <0.05 was considered statistically significant.

### Statistical analysis

The χ^2^ test was used to estimate the statistically significant difference between MUC1, EzH2 and p-p65 expression in tissues samples. Survival curve was assessed by the Kaplan-Meier method. Differences between two conditions were analyzed by the student's *t* test. In all cases, *p* < 0.05 was considered statistically significant.

## SUPPLEMENTARY MATERIALS FIGURES


