# Supplementary material for: Abnormally glycosylated MUC1 establishes a positive feedback circuit of inflammatory cytokines, mediated by NF-κB p65 and EzH2, in colitis-associated cancer
Source: Oncotarget. 2017 Oct 27;8(62):105284–98. doi: 10.18632/oncotarget.22168 (PMC5739638; doi:10.18632/oncotarget.22168)
Supplement: Supplementary file 1 [file oncotarget-08-105284-s001.pdf]

Abnormally glycosylated MUC1 establishes a positive feedback circuit of inflammatory cytokines, mediated by NF-κB p65 and EzH2, in colitis-associated cancer

SUPPLEMENTARY MATERIALS

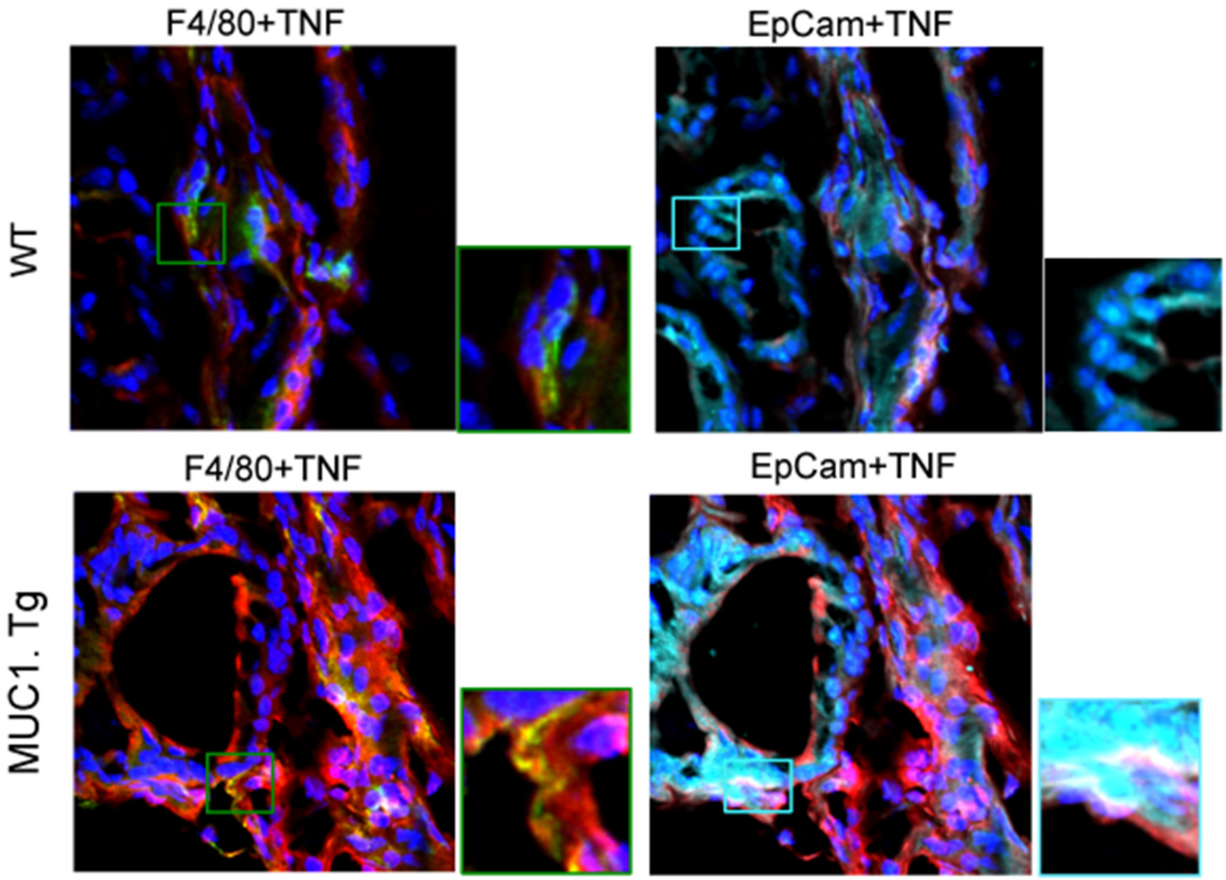

Supplementary Figure 1: TNF-α expression in AOM/DSS-treated WT and MUC1.Tg mice. Confocal immunofluorescence microscopy of frozen colon tissue samples, fixed and stained with F4/80 (green), EpCAM (cyan) and anti-TNF antibody (red). Magnification 80X. Bar: 200 µm.

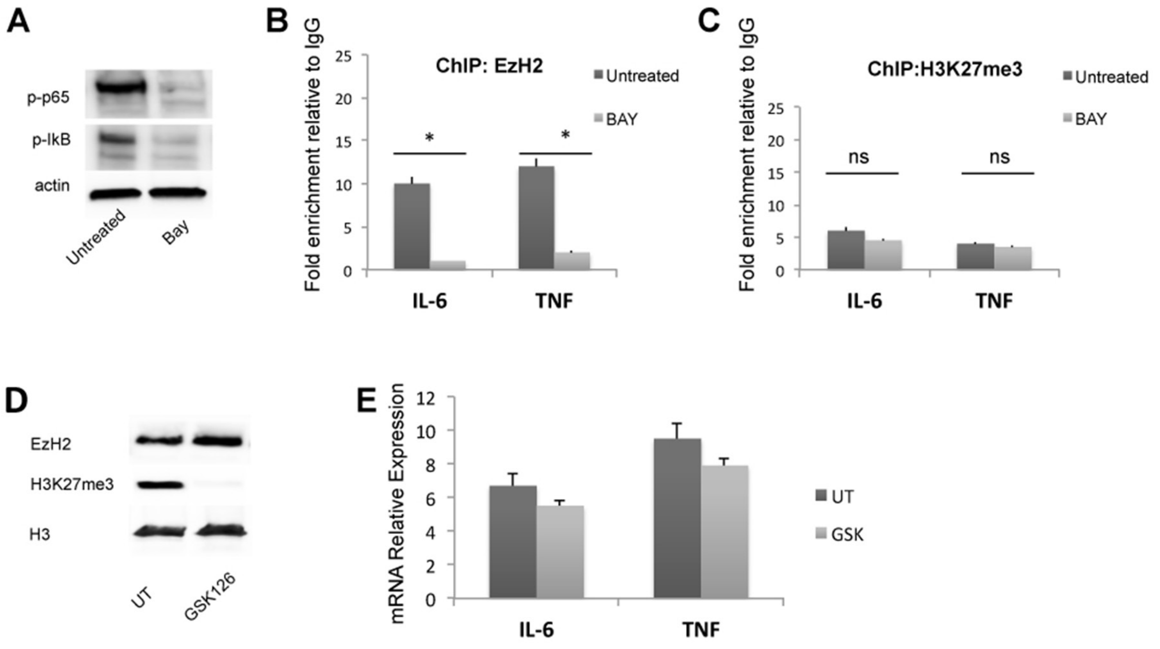

Supplementary Figure 2: Binding and regulation of the IL-6 and TNF promoters by EzH2. **(A)** Western Blotting of p-p65 and p-IκB in IECs cells treated with BAY-117085 (BAY) or left untreated. Actin was used as loading control. **(B)** ChIP assay: after treatment with BAY-117085 (BAY), soluble chromatin of Caco-2 cells was immunoprecipitated with indicated antibodies and analyzed for κB consensus sites of IL-6 and TNF-α promoters. Untreated cells were used as control. Quantification of binding was represented as fold-enrichment relative to IgG. **(C)** Western Blotting of EzH2 and H3K27me3 in MUC1+IECs cells treated with GSK-126 or left untreated. Actin was used as loading control. **(D)** mRNA relative expression levels of indicated genes MUC1+IECs cells.
